# Thinking big in small scale: characterization of peroxidase production in *Komagataella phaffii* using two-compartment scale-down system

**DOI:** 10.1007/s00449-026-03415-6

**Published:** 2026-08-28

**Authors:** Christian Mathis Wagner, Katharina Schwartz, Anton Glieder, Wolfgang Wiechert, Marco Oldiges

**Affiliations:** 1 Institute of Bio- and Geosciences IBG-1: Biotechnology, Forschungszentrum Jülich GmbH, 52425 Jülich, Germany; 2 Institute of Biotechnology, RWTH Aachen University, Worringerweg, 52074 Aachen, Germany; 3 Bisy GmbH, 8200 Hofstätten an der Raab, Austria; 4 Computational Systems Biotechnology (AVT.CSB), RWTH Aachen University, 52074 Aachen, Germany

**Keywords:** *Komagataella phaffii*, P_DF_ promoter, Scale-down system, Peroxidase production, Process robustness, Glycerol derepression

## Abstract

The replacement of cobalt-based siccatives in unsaturated polyester resin curing requires sustainable and scalable biocatalytic alternatives, with fungal peroxidases representing a promising option. For industrial implementation, however, robust large-scale production processes are essential. In this study, we investigated the robustness of a glycerol-based peroxidase production process in *K. phaffii* driven by the glycerol-derepressible P_DF_ promoter, focusing on industrially relevant bioreactor inhomogeneities. A two-compartment scale-down system was used to mimic substrate and oxygen gradients arising from insufficient mixing and mass transfer at large scale. Substrate inhomogeneity alone led to a moderate reduction in peroxidase titers (approx. 23%), indicating partial sensitivity to localized substrate oversupply. In contrast, simultaneous substrate and oxygen inhomogeneity resulted in a counterintuitive increase in peroxidase titers of approx. 13% compared to homogeneous reference conditions. This suggests that dynamic transitions between substrate limitation and surplus can positively affect peroxidase formation under P_DF_ promoter control. Based on this observation, a modified process strategy incorporating glycerol pulses during the depression phase was evaluated. While this approach demonstrated high robustness, it did not enhance productivity. Overall, the results demonstrate that the developed *K. phaffii* process is resilient to large-scale inhomogeneities and can even benefit from specific dynamic cultivation conditions.

## Introduction

The curing of unsaturated polyester resins commonly relies on cobalt-based siccatives. Because cobalt is associated with resource, health and sustainability concerns, enzymatic alternatives are of increasing interest [1–7]. Fungal peroxidases and peroxygenases can generate reactive mediator species capable of initiating resin polymerization [8–10]. In the present study, an unspecific peroxygenase from *Hypoxylon* sp. EC38 (*Hsp*UPO) was used as a model biocatalyst because of its broad oxidative activity and demonstrated applicability in polyester resin curing [10–15].

Recombinant production of *Hsp*UPO was established in *Komagataella phaffii*, a widely used host for high-cell-density cultivation and secretion of heterologous proteins [16–20]. Expression was controlled by the glycerol-derepressible P_DF_ promoter, allowing methanol-free production using glycerol as the sole carbon source [20–30]. A previously developed process comprises an initial glycerol batch, an exponential fed-batch for biomass formation, and a glycerol-limited production phase designed to support P_DF_-driven expression [56].

Transfer of such processes to larger bioreactors is associated with imperfect mixing and limited oxygen transfer. Usually, spatial separation between the point of substrate addition and aeration can lead to zones in the bioreactor with strongly varying substrate and oxygen concentrations, exposing cells to repeated environmental fluctuations [20, 31–33]. Two compartment scale-down systems provide a controlled laboratory approach for investigating such effects that are very relevant for bioprocess scale-up on process performance [31, 34, 35].

In this study, we investigated the response of the *Hsp*UPO production process to substrate inhomogeneity and to combined substrate and oxygen inhomogeneity using a two-compartment stirred-tank scale-down system. We compared biomass formation, peroxidase activity, product yield, and space-time yield with a homogeneous reference cultivation. We further examined whether the response observed under localized scale-down conditions could be reproduced by glycerol pulses during the production phase. The study therefore addresses how the type and temporal characteristics of substrate exposure affect a glycerol-based *K. phaffii* production process.

## Materials and methods

### Reagents and chemicals

Chemicals and reagents utilized in this study were sourced from Merck (Sigma-Aldrich, St. Louis, MO, USA) and ROTH (Karlsruhe, Germany).

### Strain

All experiments were performed with the commercially available *K. phaffii* strain BSY110388, supplied by bisy GmbH (Hofstätten an der Raab, Austria). The strain is based on the proprietary BSYBG11JP-HP platform, referred to by the supplier as a “heme jackpot strain”, which is optimized for the production of heme-containing recombinant proteins.

BSY110388 carries a linearized commercial pBSY5S1Z expression construct containing the gene encoding mature *Hsp*UPO under the control of the P_DF_-promoter. The construct enables methanol-independent expression under glycerol- or glucose-limited conditions.

### Cryo conservation

To preserve the strains, *K. phaffii* was cultivated in YPG medium (10 g*L^− 1^ yeast extract, 20 g*L^− 1^ peptone, 20 g*L^− 1^ glycerol) in 500 mL Erlenmeyer flasks containing 50 mL working volume. Cultures were incubated on an orbital shaker (Infors HT GmbH, Einsbach, Germany) at 250 rpm (25 mm orbital diameter) and 30 °C for 18 h. Optical density at 600 nm (OD_600_) was measured, after which cells were harvested by centrifugation at 4000 *x g* and 4 °C for 15 min (Centrifuge CT 15RE, VWR International GmbH, Darmstadt, Germany). Pellets were resuspended to an OD_600_ of 90 and mixed 1:1 with sterile 500 g*L^− 1^ glycerol. The resulting suspension was aliquoted into 2 mL cryovials and stored at −80 °C.

### Seed cultures

Seed cultures were initiated by inoculating 50 mL YPG medium with 20 µL thawed, cryopreserved *K. phaffii* cultures. Cultivation was performed in 500 mL Erlenmeyer flasks on an orbital shaker (Infors HT GmbH, Einsbach, Germany) at 250 rpm (25 mm orbital diameter) and 30 °C for 20 h.

### Bioreactor cultivations

Main cultures were conducted in a 1.7 L system comprising four glass stirred-tank reactors (STRs) equipped with two Rushton impellers each (DASGIP, Jülich, Germany). Each reactor was filled with 0.7 L of an adapted basal salt medium (BSM) based on the standard BSM and PTM_1_ formulations described in the *Pichia Fermentation Process Guidelines* [36]: 1.96 g*L^− 1^ citric acid, 0.02 g*L^− 1^ calcium dichloride dihydrate, 12.6 g*L^− 1^ diammonium sulfate, 0.5 g*L^− 1^ magnesium sulfate heptahydrate, 0.9 g*L^− 1^ potassium chloride, 1 g*L^− 1^ dipotassium hydrogen phosphate, 1 g*L^− 1^ potassium dihydrogen phosphate, 4*10^− 4^ g*L^− 1^ biotin, 20 g*L^− 1^ glycerol and 4.6 mL*L^− 1^ PTM_1_ trace salts solution (0.02 g*L^− 1^ boric acid, 0.82 g*L^− 1^ cobalt dichloride hexahydrate, 0.08 g*L^− 1^ sodium iodide, 0.2 g*L^− 1^ disodium molybdate, 6 g*L^− 1^ copper sulfate pentahydrate, 65 g*L^− 1^ iron sulfate heptahydrate, 3.36 g*L^− 1^ manganese sulfate hydrate, 20 g*L^− 1^ zinc dichloride and 2.717 mL*L^− 1^ sulfuric acid).

Cultivations were performed in fed-batch mode at 30 °C with pH maintained at 6.0 using 20% (wv^− 1^) ammonium hydroxide and 4 M phosphoric acid. pH was measured using 405-DPAS-SC-K8S/225/120 electrodes (Mettler Toledo, Gießen, Germany), and dissolved oxygen (DO) was kept above 30% using VisiFerm DO 225 optical sensors (Hamilton, Reno, NV, USA). DO control was achieved by sequentially adjusting, (i) stirring speed (400–1500 rpm), (ii) gas flow rate (42–84 nL_gas_*L_liquid_^−1^*h^− 1^) and (iii) oxygen concentration in the inlet gas (21–100%).

Cultures were inoculated to an initial OD_600_ of 0.2. After an initial batch phase on 20 g*L^− 1^ glycerol (~ 18 h), a fed-batch phase for biomass accumulation was initiated. This first fed-batch phase (FB I) was conducted with an exponential feeding strategy at half maximum growth rate with ${\rm F= F\_0^* \: e^\wedge(\mu^*t)=2.6\: [mL^*h]^\wedge(-1)^*e^\wedge(0.143^*t)}$.

for 17.33 h, feeding 200 mL BSM Feed medium containing: 500 g*L^1^ glycerol, 0.35 g*L^− 1^ calcium dichloride dihydrate, 6.45 g*L^− 1^ magnesium sulfate heptahydrate, 10 g*L^− 1^ potassium chloride, 1.2*10^− 3^ g*L^− 1^ biotin, and 15 mL*L^− 1^ PTM_1_ trace salts solution. Subsequently, the second fed-batch phase (FB II) for peroxidase production was initiated. During this phase the feed was switched to a constant feed, delivering 3.85 mL*h^− 1^ of BSM Feed medium. This feed rate corresponds to 20% of the maximum glycerol uptake rate at the end of FB I, which was empirically assessed beforehand [56]. Samples were collected at regular intervals for downstream processing and analysis.

### Scale-down cultivations

The scale down setup consists of two connected stirred tank reactors as formerly described [37], consisting of a larger 1.7 L and a smaller 0.25 L reactor STR. The two reactor compartments were connected by two tubing lines, each with a dead volume of approximately 20 mL, resulting in an approx. residence time of 14.3 min for one passage through the compartments. During cultivation, the reactor compartments were controlled individually, enabling inhomogeneous conditions as the culture was circulated between compartments with a defined residence time distribution. Cultivations were started with a 0.8 L working volume in the large compartment and the pumps were initiated after the conclusion of FB I at which point the whole cultivation volume was increased to 1 L due to feeding. From that point on, 200 mL of the cultivation suspension was contained in the small reactor compartment, and the rest of the cultivation suspension was present in the large reactor compartment. The average residence time of the cells was set to 2.85 min in the small compartment. Other process parameters were kept identical to the reference cultivation setup, only the feed rate was adjusted volumetrically proportional to the higher starting volume of 0.8 L.

### Cell dry weight determination

For cell dry weight (CDW) determination, 1.5 mL microcentrifuge tubes were pre-dried in a drying oven for 24 h, followed by storage in a desiccator for an additional 24 h, and weighed. For sampling, 1 mL culture was centrifuged at 21,000 *x g* and 4 °C for 5 min (Centrifuge CT 15RE, VWR International GmbH, Darmstadt, Germany). The supernatant was stored at −20 °C, and the pellet was dried in a drying oven for 24 h, then in a desiccator for at least 24 h, before final weighing to determine CDW.

### ABTS assay for peroxidase activity assessment

Peroxidase activity was determined using an ABTS-based assay adapted from Rotilio et al. [15]. Culture supernatants were diluted 1:100 in assay buffer (210 mM sodium acetate, pH 4.5). From the diluted sample, 20 µL were added to 180 µL substrate solution consisting of 19 mL assay buffer, 1 mL ABTS stock (17.1 mM ABTS in 50 mM sodium acetate, pH 4.5), and 7.5 µL 3% hydrogen peroxidase. Absorbance at 405 nm was recorded over 10 min in a microplate reader (Infinite M200 Pro, Tecan, Männedorf, Switzerland), with measurements taken approximately every 40 s. Enzyme activity (U*mL^− 1^) was calculated according to:

$$\:\mathrm{U}\:=\:\left({\varDelta\:\mathrm{A}\varDelta\:\mathrm{t}}^{-1}\:\mathrm{*}\:\mathrm{V}\mathrm{t}\mathrm{o}\mathrm{t}\:\mathrm{*}\:\mathrm{D}\right)\:/\:\left(\mathrm{V}\mathrm{s}\mathrm{a}\mathrm{m}\mathrm{p}\mathrm{l}\mathrm{e}\:\mathrm{*}\:{\upepsilon\:}\:\mathrm{*}\:\mathrm{d}\right)$$

$\:\mathrm{U}$ units per mL [µmol*mL^− 1^*min^− 1^].

$\:\mathrm{V}\mathrm{t}\mathrm{o}\mathrm{t}$ total reaction volume [mL].

$\:{\varDelta\:\mathrm{A}\varDelta\:\mathrm{t}}^{-1}$ change in absorption at 405 nm per time [min^− 1^].

$$\:\mathrm{D}$$ dilution factor.

$\:\mathrm{d}$ layer thickness [cm].

$\:{\mathrm{V}}_{\mathrm{s}\mathrm{a}\mathrm{m}\mathrm{p}\mathrm{l}\mathrm{e}}$ sample volume [mL].

$\:{\upepsilon\:}$ extinction coefficient at 405 nm (36) [mL*µmol^− 1^*cm^− 1^].

## Results and discussion

### Reference fed-batch process for peroxidase production

**Fig. 1:**
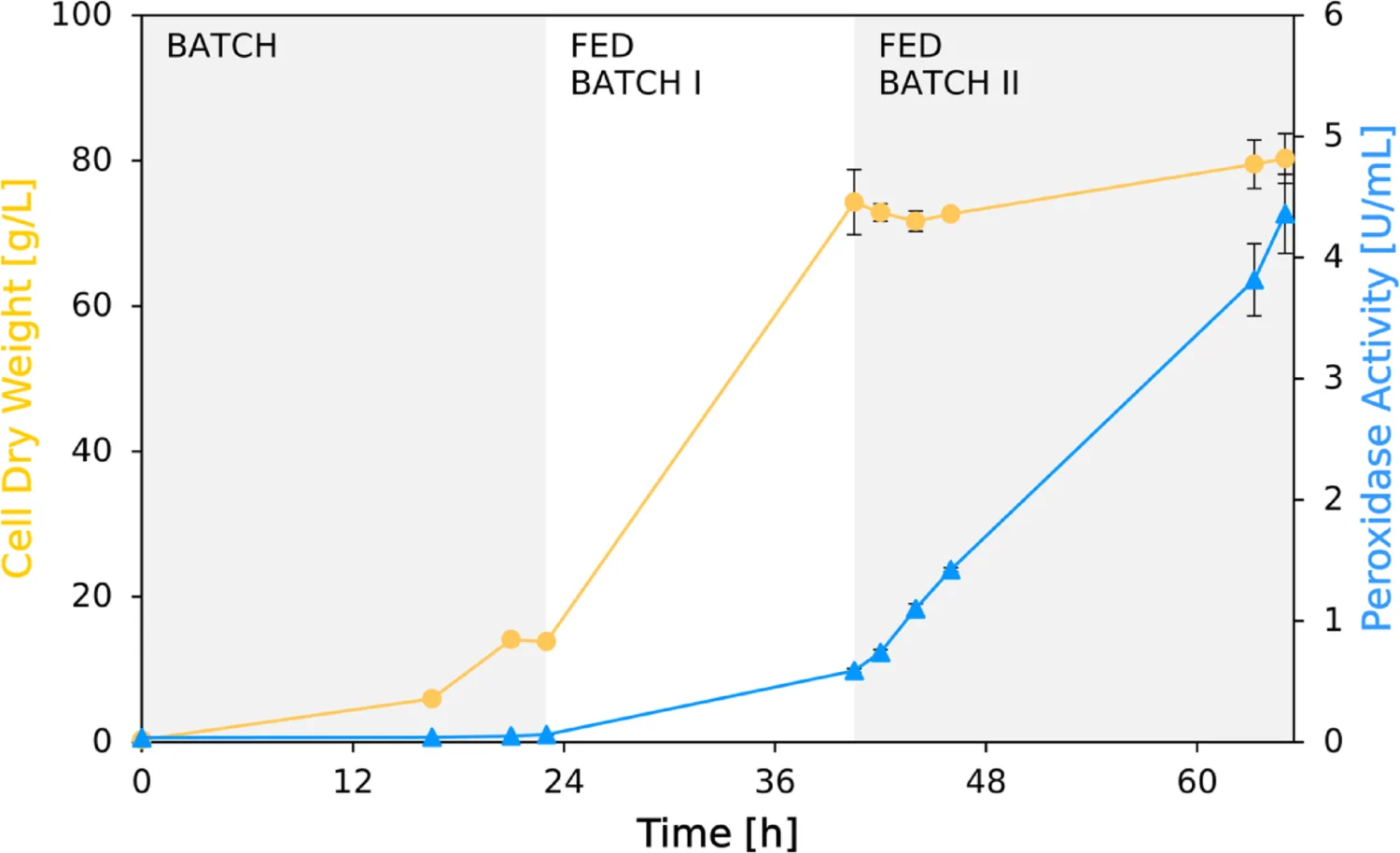
Peroxidase production process utilizing glycerol derepression (20%). Displayed are the cell dry weight and volumetric peroxidase activity over the time course of the production process. The process strategy includes an initial glycerol batch (20 g*L^−1^), followed by a first glycerol fed-batch at 5% of the maximum growth rate and second, constant fed-batch at 2% of the maximum glycerol uptake rate. The cultivations were performed with adapted basal salt medium, at 30 °C and pH = 6. (n_technical_ = 3) [56]

A process strategy for the production of peroxidases with *K. phaffii* under the control of the P_DF_ promoter has recently been established [56]. The strategy includes 3 phases: (i) an initial batch phase with 20 g*L^− 1^ of glycerol, (ii) a following fed-batch phase (FB I) with exponential feed rate of µ = 0.143 h^− 1^ at, representing 50% of the maximum specific growth rate (µ_max_), and (iii) a second fed-batch phase (FB II) with a constant feed supplying 20% of the maximum glycerol uptake rate determined at the end of phase FB I. This reduction in feed rate strongly derepresses the P_DF_ promoter and full peroxidase production is initiated (Fig. 1). However, small promotor induction seems to be present already during exponential feeding in FB I as approx. 0.6 U*mL^− 1^ peroxidase activity is measured at the end of FB I.

The process delivers consistent results, achieving a final titer of 4.36 U*mL^− 1^, yield 28.36 U*g^− 1^, and obtained a space-time yield of 67.08 U*L^− 1^*h^− 1^. It also shows robustness against impurities in crude glycerol, which can be utilized as an alternative carbon source [56].

### Scale-down bioreactor setup

**Fig. 2:**
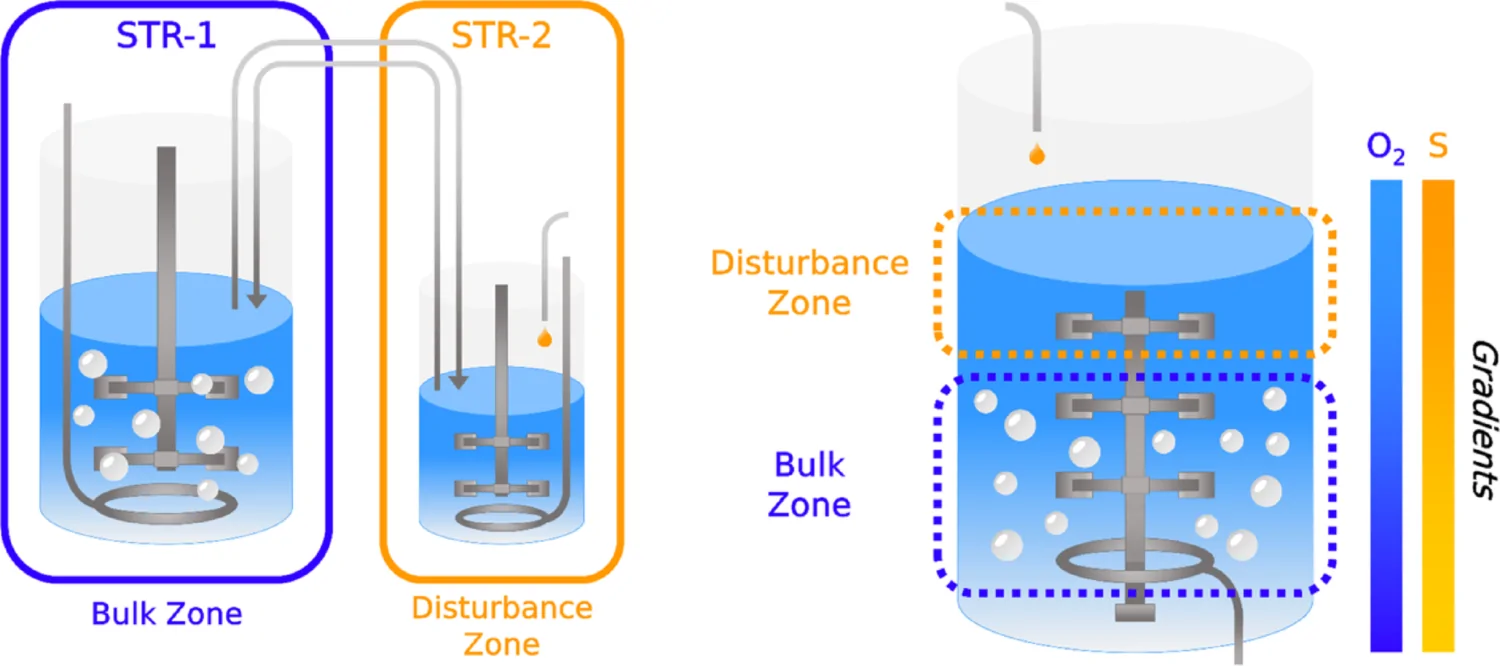
Schematic overview of the industrial scale system (right) and the scale-down system (left). The industrial scale system comprises a disturbance zone at the top (high substrate and low oxygen concentration) and a bulk zone (low substrate and high oxygen concentration). The scale-down system mimics these zones by spatial separation of the culture broth in two stirred tank reactors that can be controlled individually, with aerated, aerobic and substrate limited STR-1 and non-aerated oxygen depleted STR-2 with substrate surplus, since STR-2 receives the total volume of substrate feed for both compartments

To investigate the effects of large-scale-relevant substrate and oxygen gradients, a two-compartment stirred-tank scale-down system was used [37, 38]. STR-1 represented the aerated, substrate-limited bulk zone, whereas STR-2 represented the disturbance zone receiving the complete substrate feed and, where indicated, operated without aeration. The culture volume was distributed between the two compartments at a ratio of 80:20. In the present setup, the mean residence time in STR-2 was 2.85 min (171 s). Based on the nominal circulation flow, the corresponding theoretical mean residence time in STR-1 was approximately 11.4 min, resulting in a mean circulation period of approximately 14.3 min. These values represent population averages because the stirred-tank compartments exhibit broad residence-time distributions. The selected volume ratio and circulation rate were based on the previously characterized system of Limberg et al. [35, 37] and were chosen to reproduce residence-time ranges relevant to large-scale mixing inhomogeneities. A schematic comparison of the scale-down and industrial-scale system is provided in Fig. 2.

Such scale-down system allows spatial separation of the culture suspension in two stirred tank reactor compartments that can be controlled individually. STR-1 represents the bulk zone with aerated, aerobic and substrate limited conditions. STR-2 reflects the disturbance zone and allows different settings with, i.e., non-aerated oxygen depleted and/or substrate surplus conditions, since STR-2 receives the total volume of substrate feed for both compartments.

### Substrate inhomogeneity

**Fig. 3:**
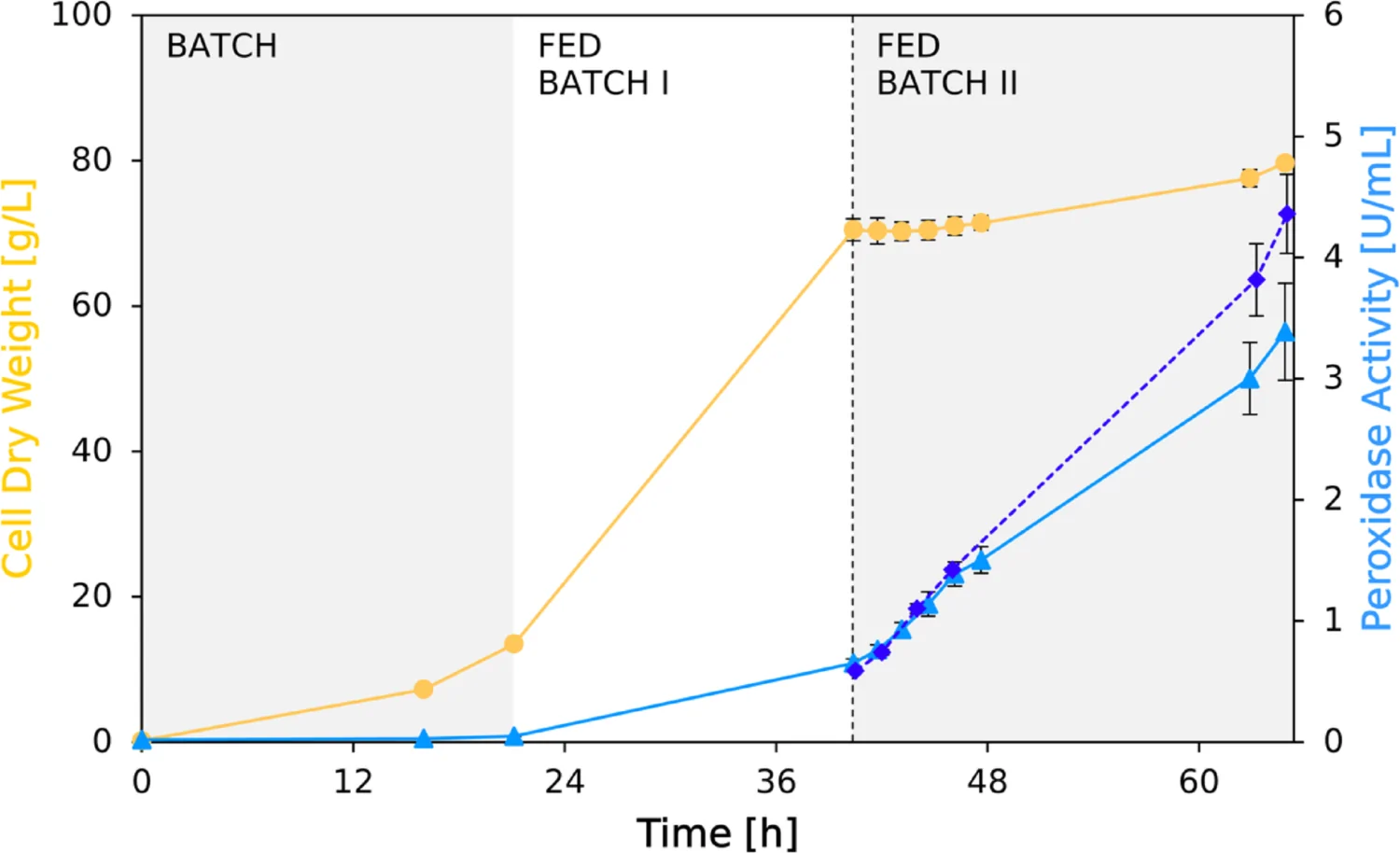
Peroxidase production process under substrate inhomogeneous conditions. Displayed are the cell dry weight (yellow) and volumetric peroxidase activity (light blue) with reference process data (dark blue, dashed) over the time course of production process. The process strategy includes an initial glycerol batch (20 g*L^− 1^), followed by a first glycerol fed-batch at 50% of the maximum growth rate and second, constant fed-batch at 20% of the maximum glycerol uptake rate. The cultivations were performed with adapted basal salt medium, at 30 °C and pH = 6. The peristaltic pumps to start the scale-down setup of the workflow were started after the first fed-batch phase and cells experienced a mean residence time of 2.85 min in the small reactor compartment with substrate surplus conditions (n_technical_ = 3)

In a first step to elucidate the effect on insufficient mixing, we examined the impact of substrate inhomogeneity. Both reactor compartments were aerated, with an oxygen cascade applied to maintain a dissolved oxygen concentration of at least 30%. In contrast, the feed solution was administered to the small reactor compartment only, representing the disturbance zone at the top of an industrial-scale system, where substrate solutions are typically introduced. Apart from this modification, all process conditions were identical to the reference process. The corresponding process data and the comparison of peroxidase activity with the reference process are shown in Fig. 3.

The progression of the cell dry weight was highly comparable to the reference process, with final values of approx. 80 g*L^− 1^. This outcome is not surprising, as biomass accumulation phases (batch and FB I) were not performed under inhomogeneous conditions and reflect reference process behavior. The time course data of the peroxidase activity in phase FB II were highly similar until 40 h, but led to reduced values under prolonged substrate inhomogeneity, resulting in a lower final titer, yield and space-time yield. While the reference process achieved a final titer of 4.36 U*mL^− 1^, the inhomogeneous process reached only a titer of 3.38 U*mL^− 1^, a yield of 21.99 U*g^− 1^, and space-time yield (STY) of 52.10 U*L^− 1^*h^− 1^ corresponding to a 22. % reduction in these key performance indicators.

Overall, substrate inhomogeneity appears to reduce process performance. Interestingly, the magnitude of this decrease roughly corresponds to the portion of cells residing in the smaller STR-2 bioreactor compartment. It could be speculated that peroxidase production in the STR-2 compartment was likely suppressed due to substrate oversupply, which was not compensated by substrate conversion in STR-1.

#### Substrate and oxygen inhomogeneity

**Fig. 4:**
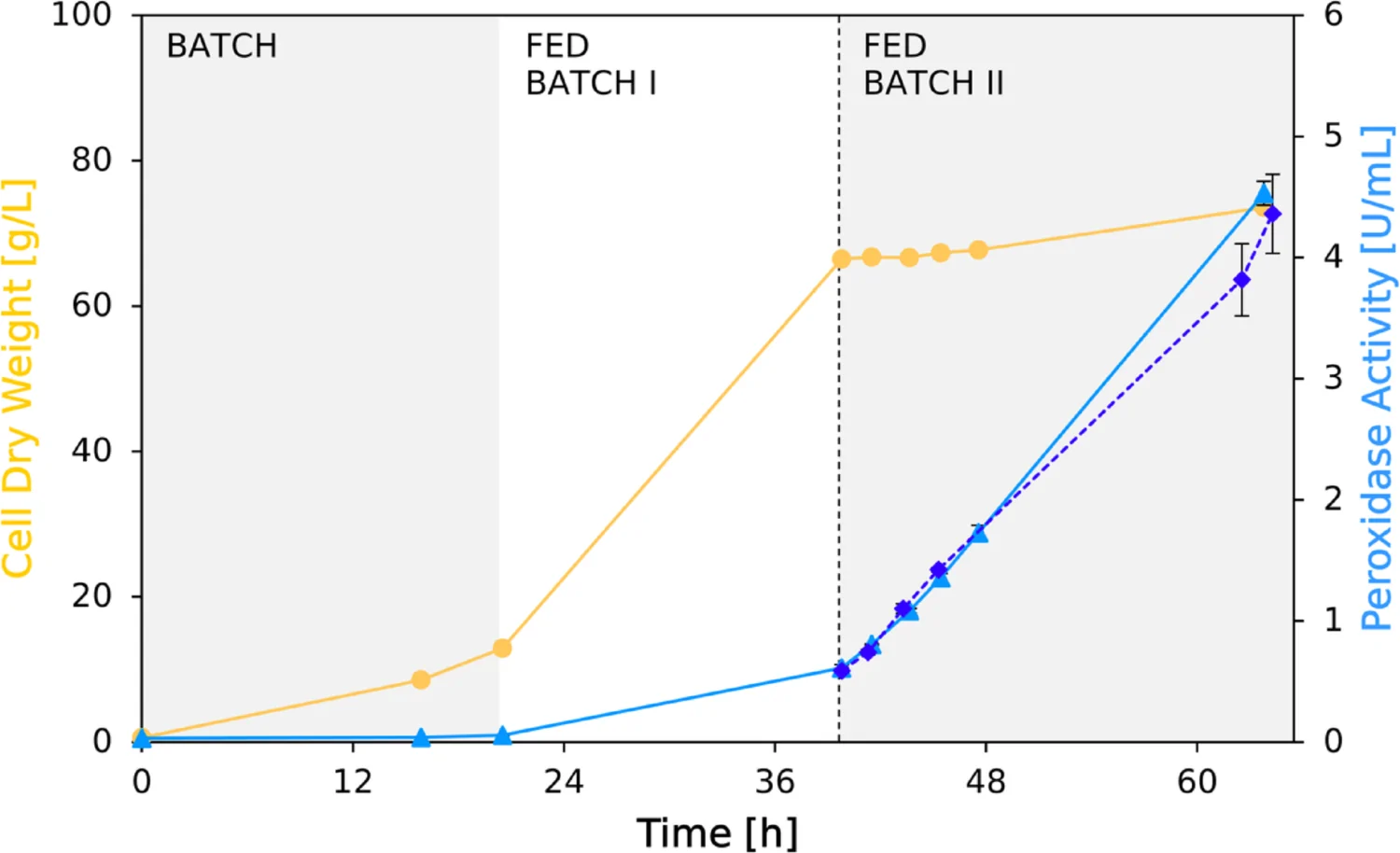
Peroxidase production process under substrate and oxygen inhomogeneous conditions. Displayed are the cell dry weight (yellow) and volumetric peroxidase activity (light blue) with reference process data (dark blue, dashed) over the time course of the production process. The process strategy includes an initial glycerol batch (20 g*L^− 1^), followed by a first glycerol fed-batch at 50% of the maximum growth rate and second, constant fed-batch at 20% of the maximum glycerol uptake rate. The cultivations were performed with adapted basal salt medium, at 30 °C and pH = 6. The peristaltic pumps to start the scale-down setup of the workflow were started after the first fed-batch phase and cells experienced a mean residence time of 2.85 min in the small reactor compartment (substrate surplus and oxygen depleted condition) (n_technical_ = 3)

After assessing the effect of substrate inhomogeneity, we next investigated the combined impact of substrate and oxygen inhomogeneity on process performance, which is the most realistic scenario in bioprocess scale-up. In industrial systems, substrate is usually introduced from the top of the reactor while aeration occurs from the bottom. To mimic these conditions, the small STR-2 reactor compartment was not aerated but received the total volume of substrate feed for both compartments leading to substrate surplus conditions with oxygen depletion at the same time. This setup reflects industrial scale inhomogeneities more realistically than substrate inhomogeneity alone. The corresponding data is presented in Fig. 4.

Strikingly, the time course data of the peroxidase activity in phase FB II were highly similar until 48 h and led to even slightly higher values under prolonged substrate and oxygen inhomogeneity, resulting in a higher final titer, yield and space-time yield. Under these conditions, a slight reduction in biomass of approximately 10% was observed, with the final cell dry weight reaching 73 g*L^− 1^. In contrast, this process achieved a higher final peroxidase titer of 4.97 U*mL^− 1^, a yield of 32.33 U*g^− 1^, and a STY of 75.58 U*L^− 1^*h^− 1^ – representing a 13% increase compared to the reference process. This outcome is noteworthy, as inhomogeneous conditions are typically judged to destabilize production and reduce process performance [31, 39].

Previous scale-down studies have reported diverse but predominantly detrimental effects of substrate and oxygen gradients on microbial production processes. Early production-scale measurements by Oosterhuis and Kossen demonstrated pronounced spatial oxygen gradients in large aerobic bioreactors, providing an important basis for subsequent scale-down investigations [33]. In *Saccharomyces cerevisiae*, substrate and oxygen fluctuations have been associated with reduced biomass formations and increased by-product formation, while bacterial scale-down studies have frequently reported reduced growth, altered metabolism, or impaired recombinant protein production under heterogeneous cultivation conditions [35, 37, 39–42]. Thus, the increase in peroxidase production observed in the present study contrasts with many previously reported responses to large-scale-relevant heterogeneities.

However, beneficial effects of environmental fluctuations are not entirely unprecedented. Moderate substrate gradients have previously been associated with increased heterologous protein productivity in recombinant *S. cerevisiae* process [43]. These contrasting observations indicate that responses to inhomogeneity depend on the organism, strain, product, promoter system, cultivation mode, and the magnitude and duration of imposed fluctuations. The present finding should therefore be regarded as specific to the investigated BSY110388 strain producing *Hsp*UPO under P_DF_ promoter control and the operating conditions of the applied two-compartment system. Because no alternative strains, products, or promoters were examined, the current data do not establish whether the effect is promoter-specific, strain-specific, product-specific, or reflects a more general physiological effect.

One mechanistically plausible interpretation of this result relates to the strongly respiratory physiology of *K. phaffii*. During aerobic growth on glycerol, the generation and reoxidation of reducing equivalents are closely coupled to mitochondrial electron transport and oxidative phosphorylation. Metabolic flux analysis of glycerol-grown *K. phaffii* showed that excess cytosolic NADH is transferred to the mitochondria and oxidized through the electron transport chain. The resulting theoretical oxygen demand accounted for more than 97% of the experimentally determined oxygen consumption [44]. Consequently, oxidative glycerol utilization and ATP generation would be expected to become transiently restricted in the non-aerated STR-2 compartment, despite the local substrate surplus. This interpretation is consistent with the lower final biomass concentration observed under combined substrate and oxygen inhomogeneity. Upon recirculation into the aerated STR-1 compartment, respiratory metabolism and ATP generation could resume. The repeated temporal separation of substrate addition from its oxidative utilization may therefore explain why combined substrate and oxygen inhomogeneity had a different effect from substrate inhomogeneity alone, under which glycerol could be metabolized in both compartments.

Oxygen limitation also affects cellular redox homeostasis more broadly. Under sustained hypoxia, *K. phaffii* shifts from fully respiratory toward respirofermentative metabolism, exhibits a reduced biomass yield, and forms arabitol, which has been proposed to contribute to the reoxidation of excess reducing equivalents [45]. Transcriptomic and metabolic studies have additionally demonstrated extensive oxygen-dependent remodeling of central carbon metabolism, ergosterol biosynthesis, stress responses, and the unfolded protein response [46]. Several studies using other recombinant proteins and expression systems have reported increased specific or volumetric productivity under controlled oxygen-limited conditions [46–48]. These findings indicate that reduced oxygen availability does not necessarily impair recombinant protein production, even though it restricts energy-efficient biomass formation. However, these studies applied sustained hypoxia and different promoters and products. The proposed mechanisms therefore cannot be transferred directly to the present process.

The regulatory properties of the P_DF_ promoter provide a further link between these physiological effects and the observed process performance. P_DF_ is a commercial variant of the *Hansenula polymorpha* formate dehydrogenase promoter that is repressed under carbon-excess conditions and strongly derepressed during carbon limitation. Its production kinetics are growth-rate dependent confirming that P_DF_-driven expression is responsive to the physiological state of the cells rather than being strictly constitutive [49]. In the present scale-down system, the absence of oxygen in STR-2 may have prevented the local glycerol surplus from being converted immediately into respiratory energy and biomass. After transfer to STR-1, the cells encountered oxygen together with glycerol originating from STR-2, but without the situation of prolonged high glycerol concentrations generated by the pulse strategy. Thus, we hypothesize that the recurring transitions between restricted glycerol utilization and renewed respiratory activity maintained a favorable balance between P_DF_ derepression, cellular energy supply, and reduced allocation of carbon to biomass. This interpretation can be seen as consistent with the simultaneous reduction in biomass and increase in product yield. Nevertheless, because compartment-specific glycerol concentrations, intracellular redox cofactors, ATP Levels, and P_DF_-driven transcript levels were not determined the proposed mechanism remains a working hypothesis that requires targeted physiological validation.

### Comparison of process KPIs and scale-down phenomena

**Fig. 5:**
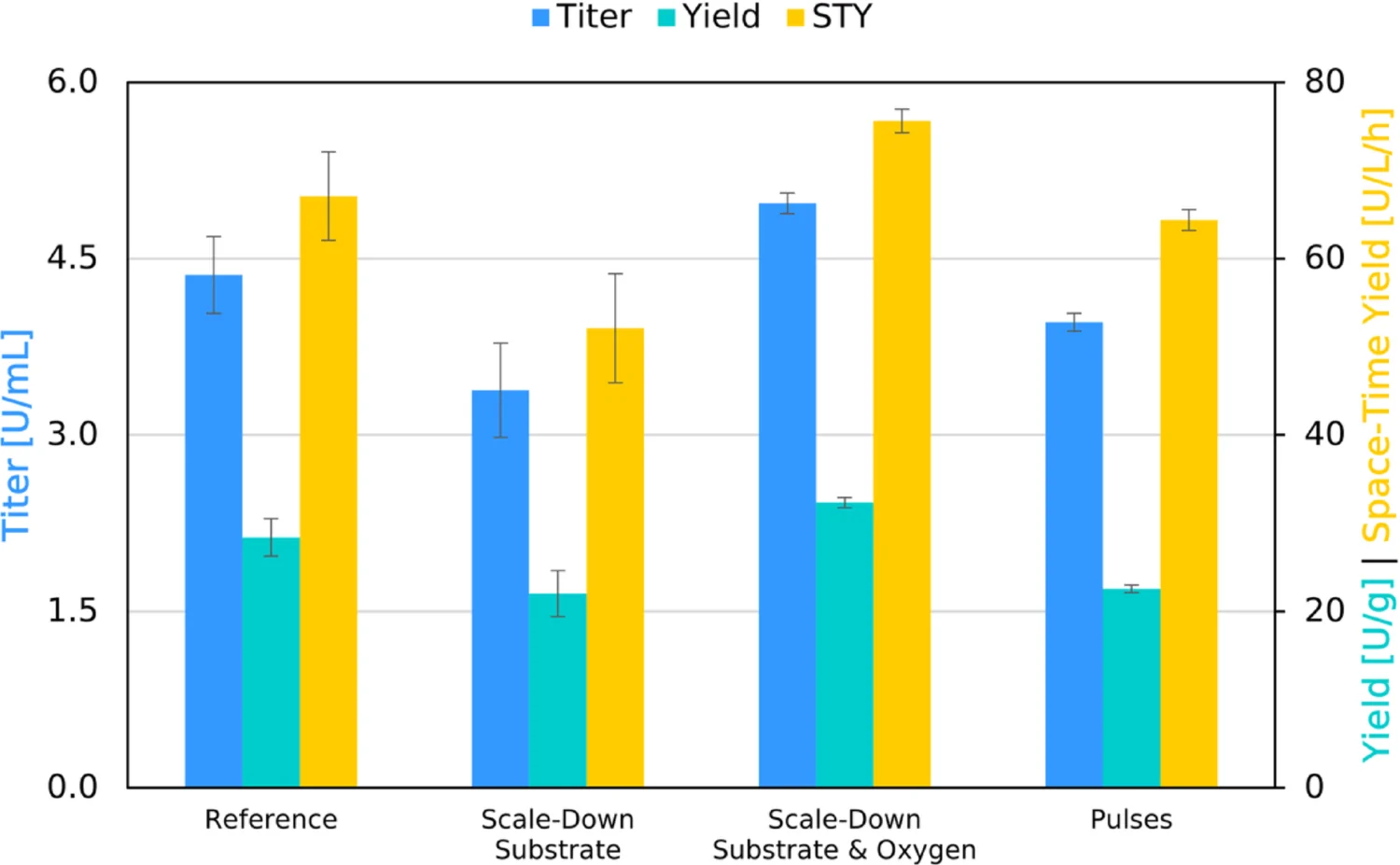
Comparisons of process key performance indicators under homogeneous reference conditions, substrate inhomogeneity, combined substrate and oxygen inhomogeneity, and glycerol-pulse conditions. Shown are final peroxidase activity, product yield, and space-time yield for each condition

To summarize the effects of inhomogeneous conditions, the key performance indicators for all tested process conditions are presented in Fig. 5.

Based on the reference process, the cultivation achieved a final titer of 4.36 U*mL^−1^, a yield of 28.37 U*g^−1^, and a STY of 67.08 U*L^−1^*h^−1^, serving as the benchmark for process performance (Fig. 5, left). Introducing substrate inhomogeneity alone resulted in a consistent reduction across all key performance indicators with a decrease of 22. % in titer (3.38 U*mL^−1^), yield (21.99 U*g^−1^), and STY (52.10 U*L^−1^*h^−1^) (Fig. 5, middle). Very strikingly, the combined presence of substrate and oxygen inhomogeneity led to an improvement in all KPIs, with increases of 14% in titer (4.97 U*mL^−1^), yield (32.34 U*g^−1^), and STY (75.59 U*L^−1^*h^−1^) (Fig. 5, right). This counterintuitive outcome suggests that the P_DF_ promoter is not adversely affected by fluctuations in substrate availability – even periodic switching between substrate depletion and surplus seems to not affect or destabilize product formation. On the contrary, such dynamic conditions may promote a more favorable induction strategy. This observation raises the question of whether the derepression phase could by further intensified by deliberately introducing controlled glycerol pulses during FB II instead of a constant feed.

### Effect of glycerol pulses on peroxidase formation

**Fig. 6:**
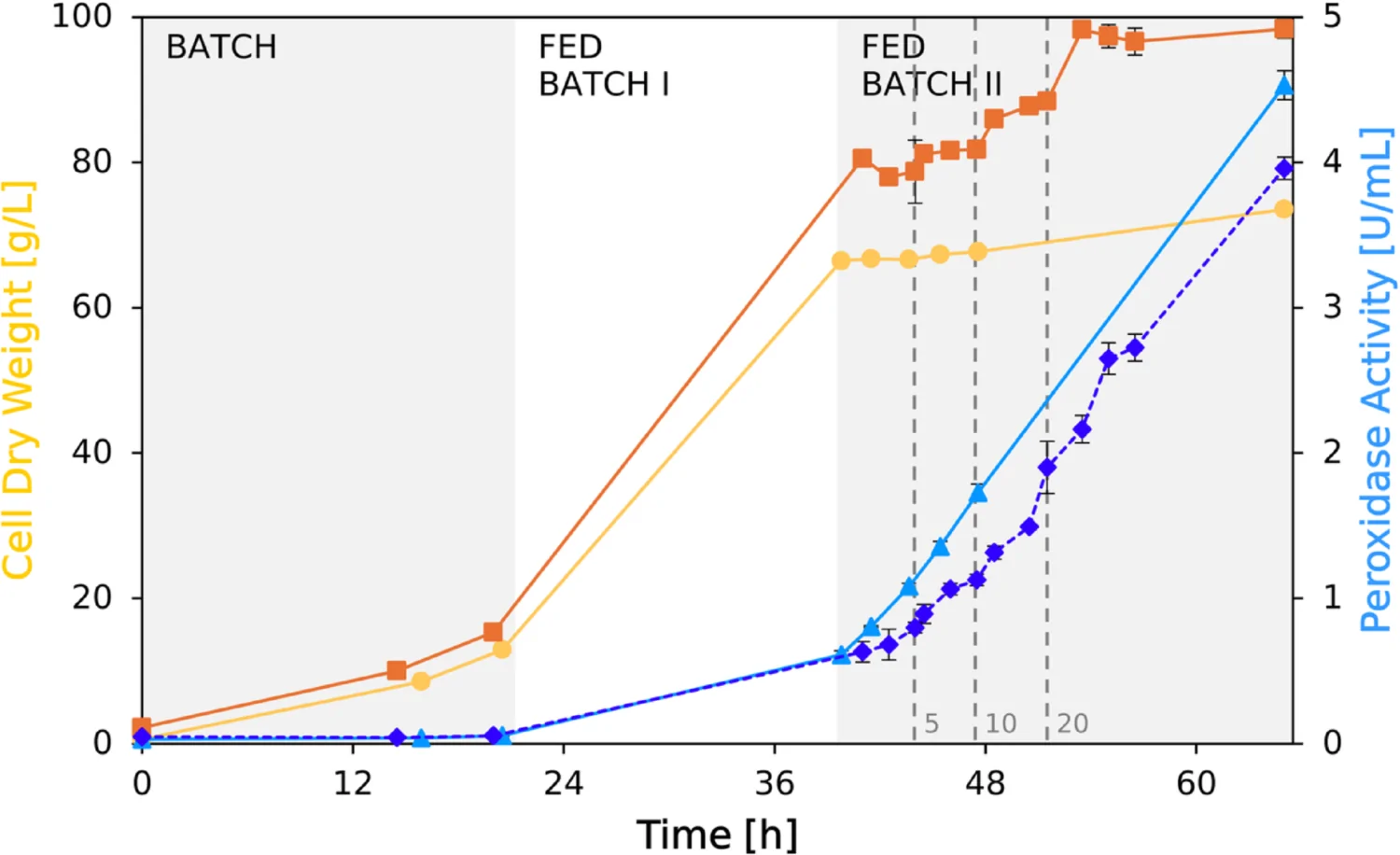
Peroxidase production under glycerol-pulse conditions during the production phase. Cell dry weight for the reference process (yellow), cell dry weight under pulse conditions (orange), volumetric peroxidase activity under pulse conditions (light blue), and the corresponding homogeneous reference-process activity (dark-blue dashed line). The process comprised an initial glycerol batch, an exponential fed-batch for biomass formation, and a second fed-batch phase in which glycerol pulses were administered at the indicated time points. Pulse amounts are shown in dark-grey dashed lines. The cultivations were performed with adapted basal salt medium, at 30 °C and pH = 6 (n_technical_ = 3)

To further investigate whether substrate availability and fluctuation could be exploited to enhance production, glycerol pulses were introduced during FB II instead of constant feed supply. This strategy was motivated by the observation that combined substrate and oxygen homogeneity led to improved key performance indicators, suggesting that periodic fluctuations in glycerol availability might positively influence induction from the P_DF_ promoter (Fig. 6).

Compared with the homogeneous reference process, glycerol pulsing during FB II resulted in lower final peroxidase activity and space-time yield despite increased biomass formation. The decrease amounted to 9.2% for the final titer (3.96 U*mL^-^1) and space-time yield (60.90 U*L^−1^*h^−1^) and 20.5% for the yield (22.56 U*g^−1^). This indicates that the imposed substrate availability did not enhance peroxidase production under these conditions. Because P_DF_ promoter activity was not measured directly, the reduced specific productivity cannot be assigned to promoter regulation alone. Notably, this reduction in productivity occurred despite a pronounced increase in biomass concentration during FB II, with the final CDW rising from 80 g*L^−1^ to 100 g*L^−1^. The decoupling of biomass formation and product formation indicates that glycerol pulsing favored growth over peroxidase production.

Importantly, the glycerol pulse experiment differed from the scale-down experiment in both the temporal and spatial characteristics of substrate exposure. During scale-down cultivation, substrate was continuously introduced into STR-2, which contained 20% of the total culture volume, and cells remained in this compartment for a mean of 2.85 min before returning to the aerobic bulk compartment. Thus, substrate enrichment was localized in STR-2 only, but transient at the population level. By contrast, the glycerol pulses increased the glycerol concentration throughout the entire culture in both compartments to values of up to 20 g*L^−1^. The pulse strategy therefore represented a spatially uniform and more prolonged substrate-excess condition rather than a direct reproduction of the short, STR-2 localized fluctuations generated by the scale-down system.

Compartment-specific glycerol concentrations and their time-course were not measured during the scale-down experiments. Accordingly, the present data support a qualitative distinction between short, localized exposure and prolonged bulk substrate excess but do not define a quantitative concentration or duration threshold separating these regimes.

The pronounced increase in final biomass concentration from approximately 80 to 100 g*L^−1^ accompanied by lower peroxidase yield and specific productivity, indicates that the glycerol pulses shifted the overall growth-production balance toward biomass formation. Such shift does not necessarily result solely from repression of the P_DF_ promoter. Recombinant protein synthesis and secretion impose substantial demands on carbon precursors, amino acids, reducing equivalents, and energy. Metabolic flux studies in *K. phaffii* have shown that recombinant protein production is accompanied by rerouting of central carbon metabolism and increased TCA-cycle and respiratory activity, reflecting the additional energetic and biosynthetic burden of product formation [50–52]. Thus, the additional glycerol supplied by the pulses may have been preferentially allocated to biomass formation and cellular maintenance rather than peroxidase synthesis.

Glycerol availability may also have altered the physiological state of the cells more broadly. In a methanol-free *K. phaffii* production system using a different promoter and product, Looser et al. observed no detectable CALB formation during growth in excess glycerol above 10 g*L^−1^, whereas product formation occurred under glycerol-limited fed-batch conditions [53]. Although these results cannot be transferred directly to the P_DF_-based process, they demonstrate that glycerol supply and growth state can strongly influence the balance between biomass and recombinant product formation. Moreover, glycerol metabolism in *K. phaffii* is closely coupled to respiratory energy generation and intracellular NADH & NADPH metabolism [44]. Abrupt increases in glycerol availability may therefore have caused transient changes in carbon flux, respiratory demand, or redox balance, even though the bulk dissolved-oxygen concentration was controlled above 30%.

A further explanation is a limitation at the level of protein folding and secretion rather than transcription. Recombinant protein production can induce the unfolded protein response in *K. phaffii*, and intracellular retention or inefficient secretion may occur when the folding and secretory capacity becomes limiting [54, 55]. These responses depend on the recombinant protein, carbon source, and physiological state of the cells. Consequently, the pulse-driven increase in growth and metabolic activity might have altered the capacity for peroxidase folding, heme incorporation, secretion, or extracellular stability. Because intracellular peroxidase, P_DF_ transcript abundance, secretion-stress markers, intracellular cofactors, and extracellular by-products were not measured, the present data cannot discriminate among altered promoter regulation, carbon allocation, metabolic imbalance, and secretion-level limitations. The reduced productivity under glycerol pulsing should therefore be interpreted as the outcome of a changed physiological state rather than as evidence for a specific P_DF_ derepression threshold [56].

## Conclusions

In this study, we systematically assessed the robustness of a glycerol-based bioprocess for heterologous peroxidase production in *K. phaffii* under control of the P_DF_ promoter, with particular emphasis on large-scale relevant inhomogeneities. Using a two-compartment scale-down system, the process was subjected to substrate inhomogeneity as well as combined substrate and oxygen inhomogeneity, thereby mimicking the disturbance and bulk zones typically encountered in industrial bioreactors.

Substrate inhomogeneity alone resulted in a clear decrease of process performance, with reductions of approx. 22.5% across all key performance indicators. This effect could be consistent with partial suppression of peroxidase production in zones experiencing substrate surplus, while biomass formation remained unchanged. In contrast, simultaneous substrate and oxygen inhomogeneity let to a counterintuitive improvement in process performance. Despite slight reduction in final biomass concentration, titer, yield and space-time yield increased by 14% compared to the homogeneous reference process. These results demonstrate that the developed process is not only resilient to realistic large-scale gradients but might even benefit from specific dynamic cultivation conditions.

Building on this observation, a modified process strategy was evaluated in which glycerol pulses were intentionally applied during the derepression phase. In contrast to the scale-down experiments, this approach did not enhance productivity. Instead, glycerol pulsing promoted additional biomass formation while reducing specific peroxidase production These findings suggest that short and frequent substrate fluctuations arising from mixing limitations are tolerated and may even be beneficial for peroxidase production, whereas prolonged or excessive glycerol availability can favor biomass formation over product formation.

Overall, the results indicate that the P_DF_-based peroxidase production process tolerates the specific substrate and oxygen inhomogeneities reproduced in the present scale-down system. These findings provide and initial indication of process robustness under selected large-scale-relevant conditions but do not establish general scalability or industrial readiness. Moreover, the data provide new insights into the process behavior of the P_DF_-based production system, suggesting that dynamic transitions between substrate limitation and surplus may favor peroxidase formation, whereas sustained glycerol excess is detrimental to specific productivity under the tested conditions. Because P_DF_ transcript levels and promoter activity were not measured directly, the present study cannot determine whether the observed changes in peroxidase production resulted from altered P_DF_ promoter regulation. The proposed regulatory interpretation therefore remains a working hypothesis and future studies should focus on elucidation of P_DF_ promotor function using, e.g., transcript and protein level investigations.

This work supports the further development of methanol-free peroxidase production processes and identifies specific inhomogeneity regimes that should be considered during scale-up. Further studies addressing oxygen-transfer capacity, by-product formation, additional spatial gradients, and long-term process stability are required to assess industrial applicability more comprehensibly. In the broader context, these findings increase the feasibility of enzymatic alternatives to cobalt-based siccatives and represent a further step toward more sustainable polymer curing technologies.

## Data Availability

Experimental data and datasets used and/or analyzed during the current study are available from the corresponding author on reasonable request.
